# Vertebral-level discrimination of incidental vertebral fractures using volumetric BMD, texture features, and finite element–derived fracture load: an exploratory study

**DOI:** 10.1007/s00256-026-05147-w

**Published:** 2026-02-16

**Authors:** Daniel Strack, Michael Dieckmeyer, Nico Sollmann, Thomas Baum, Jan S. Kirschke, Karupppasamy Subburaj

**Affiliations:** 1 Department of Mechanical and Production Engineering, Aarhus University, Katrinebjergvej 89, 8200 Aarhus N, Denmark; 2 Department of Diagnostic, Interventional, and Pediatric Radiology, Inselspital, University of Bern, Bern, Switzerland; 3 Department of Diagnostic and Interventional Radiology, University Hospital Ulm, Ulm, Germany; 4 Department of Nuclear Medicine, University Hospital Ulm, Ulm, Germany; 5 Department of Diagnostic and Interventional Neuroradiology, School of Medicine and Health, TUM Klinikum Rechts Der Isar, Technical University of Munich, Munich, Germany; 6 TUM-Neuroimaging Center, TUM Klinikum Rechts Der Isar, Technical University of Munich, Munich, Germany

**Keywords:** Fracture risk assessment, Finite element simulation, Vertebra, Texture analysis, Spine, Osteoporosis

## Abstract

**Objective:**

To assess the discriminatory ability of vertebra-specific volumetric bone mineral density (vBMD), finite element analysis-derived fracture load (FEA-derived FL), and texture analysis (TA) features for incidental vertebral fractures, and to compare performance between thoracic and lumbar levels.

**Materials and methods:**

We retrospectively reviewed baseline and follow-up thoracolumbar CT scans from 420 patients and identified 11 patients with incidental vertebral fractures contributing to 20 fractured vertebrae (7 females; mean age 65.5years). For each fractured vertebra, three level-matched control vertebrae from patients without fractures were selected, yielding 58 controls across 29 control patients (total 78 vertebrae). Parameters evaluated include vBMD, FEA-derived FL, and TA features (24 total). Discriminatory ability was assessed using area under the curve (AUC) values.

**Results:**

vBMD, FEA-derived FL, and 4 of 24 TA features showed group-wise differences between fractured and control vertebrae groups. AUCs were 0.76 [95% CI 0.55–0.90] (vBMD) and 0.73 [95% CI 0.52–0.90] (FEA-derived FL); selected texture features ranged 0.70–0.72. Region-stratified AUC point estimates were higher in the lumbar than in the thoracic vertebrae, but the 95% CIs were wide/overlapping; comparisons are descriptive.

**Conclusion:**

vBMD had the numerically largest AUC point estimate for discriminating fractured from control vertebrae; FEA-derived FL was similar, and selected texture features showed modest discrimination with comparable point estimates across lumbar and thoracic levels, generating the hypothesis of less region dependence. Regional comparisons are descriptive. Findings are exploratory and intended to prioritize candidate measures for validation and future multivariable modeling before any clinical application.

**Supplementary Information:**

The online version contains supplementary material available at 10.1007/s00256-026-05147-w.

## 1 Introduction

Osteoporosis predisposes individuals to an elevated risk of vertebral fractures, affecting up to one in three women and one in five men over 50 years of age [1]. As the global population ages, the incidence of osteoporotic vertebral fractures is projected to rise, underscoring the need for accessible and accurate risk assessment strategies [2]. Early identification of patients at high fracture risk is essential for targeted intervention, reduced mortality, and lower healthcare costs [3].

Currently, the diagnosis of osteoporosis and fracture risk assessment relies primarily on areal bone mineral density (BMD) derived from dual-energy X-ray absorptiometry (DXA) at the hip/femur and the Fracture Risk Assessment Tool (FRAX) [4]. Yet, these approaches can fail to identify individuals who subsequently sustain fractures [5, 6, 10, 11]. Notably, femoral DXA and FRAX stratify population hip fracture risk well, including a report that a majority of hip fragility fractures can be anticipated by femoral DXA in older adults [12]. For vertebral fractures specifically, the proportion of patients who are not classified as osteoporotic by DXA depends on how fractures are defined and ascertained, as well as the DXA site used for classification, with reports varying across populations [10, 11, 13, 14]. This diagnostic gap highlights the need for more robust risk stratification and discrimination methods and measures beyond DXA-derived BMD values [4]. Our work focuses on vertebra-level risk from routine CT scans and is not intended to replace femoral DXA for the discrimination of hip fractures.

Finite element analysis (FEA) has been adapted as a computational approach to estimate vertebral strength and fracture risk using medical imaging data [15, 16]. Ex vivo studies have demonstrated strong correlations between FEA-derived metrics and experimentally measured vertebral strength [17–19]. Subsequent studies have retrospectively investigated whether FEA-derived measurements can outperform BMD in fracture discrimination [20–25]. However, many of these studies focused on aggregated lumbar metrics [20–24] or the thoracic vertebrae [23], and some included limited female representation [22]. Dieckmeyer et al. reported no significant improvements in discrimination ability over BMD using FEA and texture analysis (TA), yet highlighted the need for vertebra-specific evaluations [20].

Considering vertebral fractures manifest across multiple spine levels, this study evaluated fracture risk parameters at the individual vertebral level. The primary objective was to compare the discriminatory ability of volumetric BMD (vBMD), FEA-derived fracture load (FL), and CT-based texture features for incidental vertebral fractures. The secondary objective was to descriptively explore whether discrimination differs between lumbar and thoracic vertebrae; given the small number of fracture patients and within-patient clustering, this analysis is exploratory and hypothesis-generating.

## 2 Methods

### 2.1 Study population

This study was approved by the institutional review board, and the requirement for written informed consent was waived due to the study’s retrospective design. We retrospectively identified 420 patients with clinical routine baseline and follow-up thoraco-lumbar computed tomography (CT) examinations from the institutional Picture Archiving and Communication System (PACS). All baseline and follow-up CT scans covered the thoraco-lumbar spine with sagittal reformations, and analyses were restricted to vertebrae consistently visualized from T6 through L4. The exclusion criteria were as follows: (1) known history of intake of bone-active medications (e.g., bisphosphonates), (2) presence of bone metastases, and (3) known hematological or metabolic bone disorders aside from osteopenia/osteoporosis.

Nineteen of these 420 patients exhibited new incidental vertebral fractures on follow-up scans (median follow-up time, 16.25 months; interquartile range (IQR), 10.25 months). Eight of these 19 cases were excluded due to either prevalent fractures at baseline (*n* = 2) or insufficient image quality and segmentation (*n* = 6); two of these six cases also had prevalent fractures at baseline but were counted only once under the image quality exclusion criterion. The final cohort included 11 patients (7 female, mean age: 65.5 years, standard deviation (SD): 9.7, mean age female 69.0 years (range 60–79), mean age male 59.3 years (range 41–67)) with a total of 20 vertebral fractures distributed across the thoracic (T6–T12, *n* = 11) and lumbar (L1–L4, *n* = 9) spine regions. Patients could contribute multiple fractured vertebrae at different levels; however, by anatomy, a patient cannot fracture the same vertebral level twice. Baseline and follow-up CT examinations were obtained as part of routine clinical care for indications unrelated to osteoporosis assessment (oncologic staging/surveillance, to rule out tumor recurrence). Vertebrae with radiologic suspicion of metastatic involvement were excluded. Tumor site and oncologic treatment details were not consistently available.

**Fig. 1:**
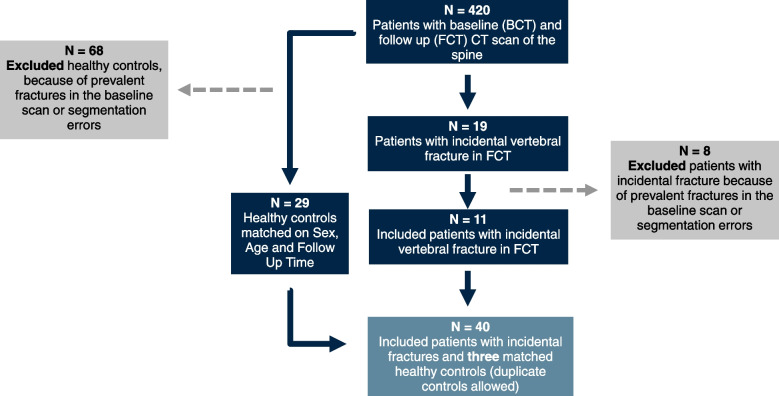
Flowchart of patient selection and inclusion criteria

For each fractured vertebra at level *v* (T6–L4), three fracture-free control vertebrae were selected at the same anatomic level *v* from different patients without prevalent or incident fractures. Matching was exact on the vertebral level and sex, and aligned on age and follow-up interval. When three distinct controls were unavailable within a stratum, we permitted duplicate matching to preserve matching stringency (i.e., the same control vertebra could be matched to more than one case); this was the case for two control vertebrae. The fracture-free control vertebrae were drawn from a sub-cohort that had no evidence of prevalent or incidental fractures in both baseline and follow-up CT scans. The final dataset, therefore, comprised 78 vertebrae (20 fractured and 58 controls). The inclusion and exclusion workflow is summarized in Fig. 1.

### 2.2 Image acquisition

All imaging examinations were conducted on a single multi-detector CT (MDCT) system (Somatom Sensation Cardiac 64; Siemens Medical Solutions, Erlangen, Bavaria, Germany). Scanning parameters included 120 kVp, automated tube-current modulation (mean effective tube load ≈ 200 mAs), and 64 × 0.6 mm collimation. Multiplanar sagittal reformats of the spine were reconstructed at 3-mm slice thickness using the standard bone kernel of the manufacturer. A non-ionic iodinated contrast agent (Imeron 400; Bracco Imaging, Konstanz, Germany) was administered intravenously at a rate of 3 mL/s using a dual-head power injector (Pilot C; Fresenius Kabi, Bad Homburg, Germany), with a 70-s acquisition delay. Contrast volume was weight adjusted: 80 mL (≤ 80 kg), 90 mL (81–100 kg), and 100 mL (> 100 kg). Additionally, all patients ingested 1000 mL of oral barium suspension (Barilux Scan; Sanochemia Diagnostics, Neuss, Germany). All baseline and follow-up CT scans were reviewed on sagittal multiplanar reformations by a board-certified neuroradiologist (TB), who was blinded to the clinical data. Vertebral fractures were graded according to the Genant semi-quantitative method [26] using height-loss thresholds: grade 1 (mild), 20–25%; grade 2 (moderate), 26–40%; and grade 3 (severe), ≥ 40%. Numeric percentage values used for grading were not retained in routine clinical practice. Anterior height loss observed in wedge deformities was calculated as the ratio of the anterior and posterior height [26]. Numerical height measurements (Supplementary Table S1) of the fractured vertebrae are reported based on readings by NS (board-certified neuroradiologist). All measurements were performed in ITK-Snap (version 3.8.0) on post-fracture scans. On sagittal CT reconstructions, linear measurements included (i) the maximum vertebral body height along the posterior margin (reference height) and (ii) the minimum vertebral body height identified on the sagittal slice demonstrating the greatest deformity. In this context, none of the registered vertebral fractures seemed to affect the posterior margin of the vertebrae (i.e., no visually detected height loss at the posterior margin, no vertebral body fractures with concomitance of their trailing edge [in the direction of the spinal cord]).

### 2.3 BMD calculation

Volumetric BMD (vBMD) values were derived from the baseline MDCT scans. Vertebral segmentation and labeling were performed automatically using SpineQ software (version 1.0; Bonescreen GmbH, Munich, Germany), which utilizes a segmentation framework consisting of convolutional neural networks (CNNs) [27]. Regarding segmentations for vBMD calculations, a mask of the trabecular compartment of the vertebral bodies was generated using asynchronous calibration (by converting Hounsfield Units [HU] to vBMD using kVp and scanner-specific equations). The scanner-specific calibration factor was previously obtained using phantom measurements (QSA-717 phantom; QRM Quality Assurance in Radiology and Medicine GmbH, Möhrendorf, Germany). SpineQ also automatically detected and corrected for the contrast media phase to mitigate potential contrast medium-induced variability in BMD estimation [28].

### 2.4 Finite element simulation

**Fig. 2:**
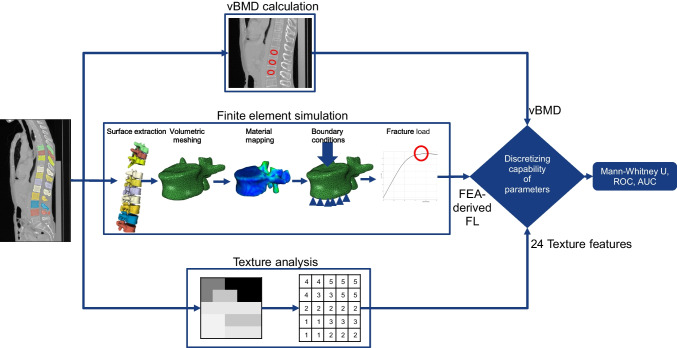
Schematic representation of the process to obtain the different parameters and the overall study design

The FE models were generated from baseline CT data using a previously validated pipeline Fig. 2 [17, 20, 29]. Briefly, vertebral bodies were delineated, reconstructed as three-dimensional (3D) geometric models, converted into subject-specific tetrahedral meshes, assigned density-dependent material properties, and simulated axial compression loading to calculate the FEA-derived FL [29].

Vertebral segmentation masks, including posterior elements, were taken from the processed CT data using SpineQ software (version 1.0; Bonescreen GmbH, Munich, Germany). These masks were inspected and post-processed. Triangulated surfaces were remeshed to four-node tetrahedral volumes (target edge length ≈ 1.25 mm) to capture the bone morphology with adequate numerical fidelity. To represent the heterogeneity of bone, individual material parameters were assigned to the mesh based on the HU using the open-source software tool Bonemat (version 4, www.bonemat.org) [30]. Elements with similar material properties were aggregated into 50 material bins via adaptive clustering to reduce computational complexity and cost [29]. Non-linear, anisotropic material properties were assigned to the resulting mesh using the equations given in Table 1.

**Table 1:** Equations to calculate material properties based on image intensities

Parameter	Relationship
**Apparent density (** ${{\boldsymbol{\rho}}}_{{\boldsymbol{a}}{\boldsymbol{p}}{\boldsymbol{p}}} {\boldsymbol{\, i}}{\boldsymbol{n\, }} {\boldsymbol{K}}{\boldsymbol{g}}/{{\boldsymbol{m}}}^{3}$)	${\rho }_{app}=47+1.122\times HU$; [45]
**Ash density (** ${{\boldsymbol{\rho}}}_{{\boldsymbol{a}}{\boldsymbol{s}}{\boldsymbol{h}}} {\boldsymbol{\,i}}{\boldsymbol{n\,}} {\boldsymbol{K}}{\boldsymbol{g}}/{{\boldsymbol{m}}}^{3}$)	${\rho }_{ash}=0.6\times {\rho }_{app}$; [46]
**Young’s modulus (** ${\boldsymbol{E}} {\boldsymbol{\,i}}{\boldsymbol{n\,}} {\boldsymbol{M}}{\boldsymbol{P}}{\boldsymbol{a}}$**)**	${E}_{z}=4730\times {\left({\rho }_{app}\right)}^{1.56}$; [47]
	${E}_{x}={E}_{y}=0.333\times {E}_{z}$; [19]
**Poisson ratio (** ${\boldsymbol{V}}$**)**	${V}_{xy}=0.381$; [19]
	${V}_{xz}={V}_{yz}=0.104$; [19]
**Shear modulus (** ${\boldsymbol{G}} {\boldsymbol{\,i}}{\boldsymbol{n\,}} {\boldsymbol{M}}{\boldsymbol{P}}{\boldsymbol{a}}$**)**	${G}_{xy}=0.121\times {E}_{z}$; [19]
	${G}_{xz}={G}_{yz}=0.157\times {E}_{z}$; [18]
**Maximum principal stress limit (** ${\boldsymbol{\sigma}}{\boldsymbol{\,i}}{\boldsymbol{n\,}} {\boldsymbol{M}}{\boldsymbol{P}}{\boldsymbol{a}}$**)**	$\sigma =137\times {\rho }_{ash}^{1.88}, {\rho }_{ash}<0.317;$[48]
	$\sigma =114\times {\rho }_{ash}^{1.72}, {\rho }_{ash}>0.317$; [48]
**Plastic strain (** ${{\boldsymbol{\epsilon}}}_{{\boldsymbol{A}}{\boldsymbol{B}}}$**)**	${\epsilon }_{AB}=-0.00315+0.0728\times {\rho }_{ash}$; [49]
**Minimum principal stress limit (** ${{\boldsymbol{\sigma}}}_{{\boldsymbol{m}}{\boldsymbol{i}}{\boldsymbol{n}}} {\boldsymbol{\,i}}{\boldsymbol{n\,}} {\boldsymbol{M}}{\boldsymbol{P}}{\boldsymbol{a}}$**)**	${\sigma }_{min}=65.1\times {\rho }_{ash}^{1.93}$; [49]
**Plastic modulus (** ${{\boldsymbol{E}}}_{{\boldsymbol{p}}} {\boldsymbol{\,i}}{\boldsymbol{n\,}}\boldsymbol{ }{\boldsymbol{M}}{\boldsymbol{P}}{\boldsymbol{a}}$**)**	${E}_{p}=-4000\times {\rho }_{ash}^{2.05}$; [49]

The material-mapped meshes were imported into and solved using a commercial FE solver, Abaqus (version 2021, Dassault Systèmes, Johnston, RI, USA). Boundary conditions included fixation of the inferior endplate (translations and rotations) and axial displacement of the superior endplate, simulating experimental compression tests used to validate FE models. The peak force from the force–displacement curve was recorded as the FEA-derived FL representing the vertebral axial strength.

### 2.5 Texture analysis

Texture analysis (TA) quantifies grayscale heterogeneity within segmented bone regions of interest. Prior to feature extraction, image intensities were normalized to a [0, 1] scale to reduce sparseness, and volumes were interpolated using cubic interpolation to ensure isotropy. A detailed description of the methodology is provided in the reference study [20]. Three categories of TA features were extracted:

- First-order features: derived from voxel intensity histograms, including skewness, variance, and kurtosis [31].
- Second-order features: derived from grey-level co-occurrence matrices (GLCM), which capture the frequency of voxel pairs with specific intensity values and spatial offsets [32].
- Higher-order features: derived from grey-level run-length matrices (GLRLM), which measure the distribution and length of contiguous voxels with identical intensities [33].

The TA was implemented and performed in the MATLAB environment (R2021a; MathWorks Inc., Natick, MA, USA), utilizing a custom-modified version of the open-source radiomics toolbox available at (https://github.com/mvallieres/radiomics) [34]. Definitions of all extracted TA features are provided in Table 2.

**Table 2:** Description of texture analysis features

Category and texture feature	Description
**First order (histogram) **[31]	
Variance	Spread of gray-level distribution
Skewness	Shape of gray-level distribution
Kurtosis	Flatness of gray-level distribution
**Second order (GLCM) **[32]	
Energy	Uniformity
Contrast	Entropy
Entropy	Randomness
Homogeneity	Homogeneous scene
Correlation	Linear spatial relationships between texture elements
Sum average	Spread of the mean voxel co-occurrence distribution
Variance	Voxel co-occurrence distribution
Dissimilarity	Heterogeneity
**Higher order (GLRLM) **[50–53]	
SRE	Short-run distribution
LRE	Long-run distribution
GLN	Similarities of the gray level
RLN	Similarities in length of runs
RP	Distribution and homogeneity of runs with a specific direction
LGLRE	Distribution of low-gray level values
HGLRE	Distribution of high-gray-level values
SRLGE	Joint distribution of short runs and low gray-level values
SRHGE	Joint distribution of short runs and high gray-level values
LRLGE	Joint distribution of long runs and low gray-level values
LRHGE	Joint distribution of long runs and high gray-level values
GLV	Weighted variances of gray-level runs
RLV	Weighted variances of gray-level runs

Although routine MDCT cannot resolve individual trabeculae, TA features can summarize spatial gray-level patterns across multiple voxels that arise from the mixture of bone matrix and bone marrow, as well as from the coarse architectural organization. Features capturing gray-level heterogeneity and gray-level co-occurrence matrix (GLCM) statistics (e.g., contrast, variance, entropy) and run-length statistics (e.g., short-run and long-run emphasis) can reflect differences in apparent trabecular spacing, connectivity, and disorganization, thus properties linked to mechanical competence. Prior work has shown that MDCT-derived TA features correlate with FEA-derived FL and that combining texture with vBMD improves fracture discrimination [35, 36].

### 2.6 Statistical analysis

All statistical analyses were performed in Python (version 3.11, https://www.python.org/) using the SciPy library (version 1.11.1, https://scipy.org/). Given the modest number of fracture patients in this exploratory study and events-per-variable considerations, analyses were prespecified as univariable and target discrimination at the vertebra level, quantified by the area under the ROC curve (AUC). Because multiple vertebrae may arise from the same patient, AUCs were computed with 95% confidence intervals obtained by patient-clustered bootstrap (2000 iterations). No formal pairwise AUC tests were performed. Group-wise (controls vs. fracture vertebrae) summaries (medians with interquartile ranges) are also provided to describe the cohort. Twenty-six prespecified parameters were evaluated univariately: vBMD, FEA-derived FL, and 24 TA features. To correct for multiple comparisons, the Benjamini–Hochberg procedure was applied with a false discovery rate (FDR) of 5% [20, 37]. Variables with *q* < 0.05 are flagged for downstream analysis. Discriminatory abilities of vBMD, TA features, and FEA-derived FL were quantified using receiver operating characteristic curve (ROC) analysis, with the area under the curve (AUC) serving as the primary metric. To facilitate clinical interpretation, ROC analyses were stratified by region (thoracic vs. lumbar) and contextualized using established lumbar vBMD thresholds of 80/120 mg/mL (osteoporosis/osteopenia) [38] and the proposed thoracic cutoff of 100 mg/mL (Rühling et al. [39]). Given the limited sample size (and few fractures per region), region-stratified ROC/AUC results are presented for illustrative, hypothesis-generating purposes only. Established lumbar thresholds are defined for L1–L3. In the absence of other thresholds, we applied the lumbar cut points to other lumbar levels for contextual comparison only and did not use them for diagnostic classification.

## 3 Results

A total of 78 vertebrae were analyzed, comprising 20 incident fractures and 58 matched controls. Results are reported in three parts: (i) clinical and morphologic characteristics of the fracture cohort, (ii) descriptive statistics and groupwise comparisons of imaging-derived parameters, and (iii) ROC analysis of discriminatory performance of each parameter.

### 3.1 Patient and fracture characteristics

Among the eleven included patients with incidental vertebral fractures, three sustained two fractures, and three sustained three fractures, for a total of 20 fractures. These fractures were distributed across the following levels: T6, T7, T8, T10, T11 (× 3), T12 (× 4), L1 (× 4), L2 (× 3), L3, and L4. According to the Genant semi-quantitative grading scale [26], 60% of fractures were classified as grade 1, 30% as grade 2, and 10% as grade 3.

### 3.2 Descriptive statistics

Cohort characteristics are summarized in Table 3 as median [IQR]. There were no differences in age or inter-scan interval observed between the fracture and control vertebrae groups. Age alignment was close, with a maximum absolute age difference of 2.27 years across the three matched controls for each fractured vertebra. Fractured vertebrae exhibited lower vBMD and FEA-derived fracture load than controls. Among the 24-texture analysis (TA) features, four showed group-wise differences between fractured and control vertebrae: gray-level non-uniformity (GLN) was higher in fractured vertebrae, whereas high-gray-level run emphasis (HGLRE), short-run high-gray-level emphasis (SRHGLE), and long-run high-gray-level emphasis (LRHGLE) were not.

**Table 3:** Descriptive statistics for demographic, biomechanical, and image-derived texture features between fracture and control groups. Data are presented as median and IQR. Group differences were assessed using Mann–Whitney U tests, and the results were adjusted for multiple comparisons using the Benjamini–Hochberg false discovery rate (FDR) procedure at a 5% threshold. Statistically significant results (*q* < 0.05) are indicated in bold

Parameters	Fracture group		Control group		*q*-value
	Median	IQR	Median	IQR	
Age (years)	67	8	67	6,5	0.950
Follow-up (months)	23	10.25	23	16	0.937
**BMD**	86.4	47.83	127.30	49.48	**0.017**
**FEA-derived FL [N]**	2806.41	1210.14	3262.89	1691.87	**0.022**
Variance	55.14	11.29	50.91	16.99	0.874
Skewness	1.07	0.51	0.95	0.96	0.804
Kurtosis	2.75	1.92	1.77	4.17	0.472
Energy [10^–5^]	8.79	4.43	6.58	3.92	0.071
Contrast	1189.04	532.66	1510.77	857.95	0.114
Entropy	14.09	0.63	14.43	0.77	0.108
Homogeneity	0.04	0.01	0.04	0.01	0.131
Correlation	0.62	0.15	0.69	0.12	0.165
Sum Average [10^–4^]	6.66	5.31	5.97	6.01	0.638
Variance [10^–3^]	7.64	3.34E-01	9.35	6.18	0.472
Dissimilarity	25.50	6.13	28.64	8.75	0.131
SRE	0.99	2.21E-03	0.99	2.71E-03	0.131
LRE	1.04	9.21E-03	1.04	1.15E-02	0.131
**GLN** [10^–3^]	7.21	1.75	6.14	2.09	**0.042**
RLN	0.97	5.72E-03	0.98	7.02E-03	0.131
RP	0.99	2.94E-03	0.99	3.63E-03	0.131
LGLRE	1.52E-04	5.28E-05	1.53E-04	7.72E-05	1.000
**HGLRE**	18,360.99	7841.18	27,481.15	16,007.38	**0.022**
SRLGLE	1.51E-04	5.25E-05	1.52E-04	7.64E-05	1.000
**SRHGLE**	18,210.52	7835.89	27,314.35	15,934.81	**0.022**
LRLGLE [10^–4^]	1.56	5.42E-01	1.56	8.21E-01	0.982
**LRHGLE**	18,979.72	7856.26	28,224.87	16,274.47	**0.022**
GLV	0.05	0.03	0.06	0.04	0.874
RLV [10^–6^]	3.25	2.04	2.53	2.26	0.165

### 3.3 ROC analysis

**Fig. 3:**
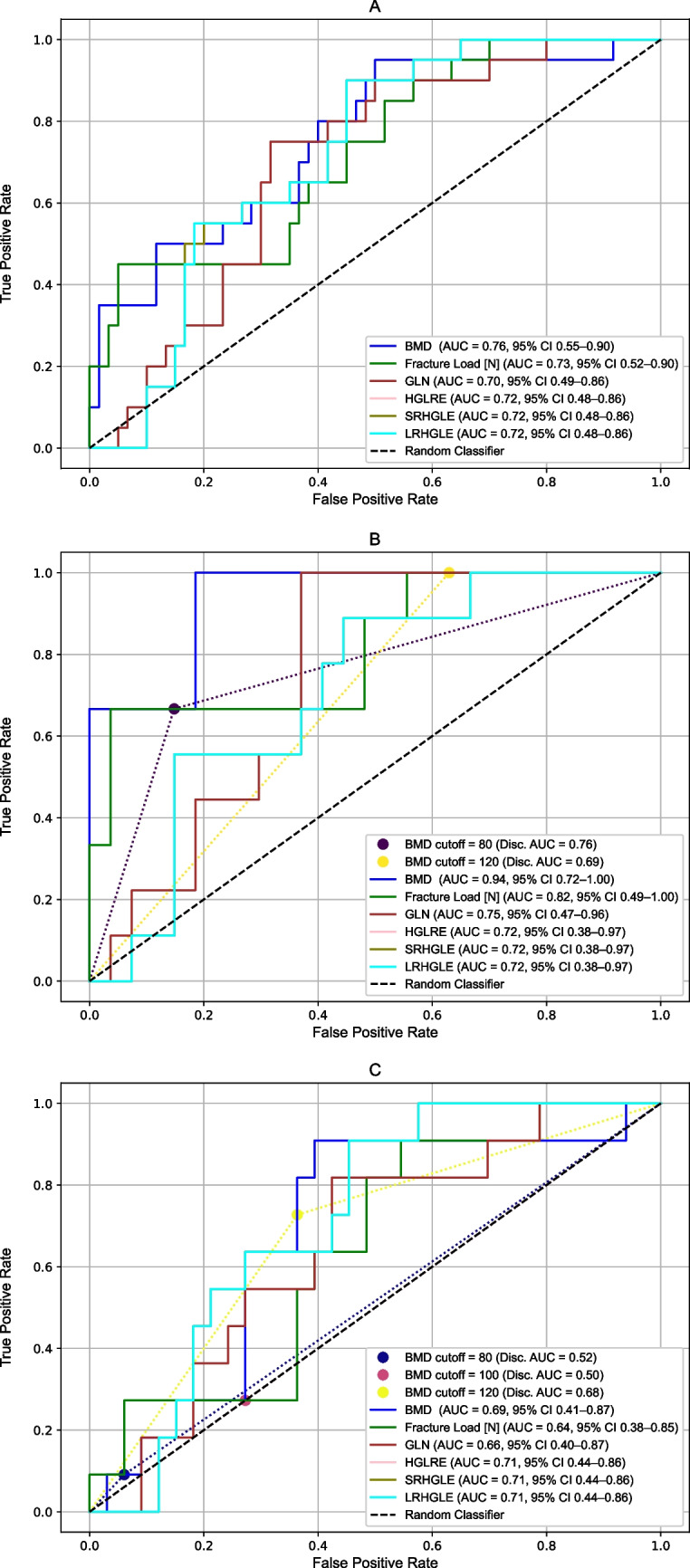
Receiver operating curve and area under the curve with 95% CI calculated for **a** the complete dataset, **b** only the lumbar vertebrae, **c** only the thoracic vertebrae

Discrimination is reported as AUC with patient-clustered 95% confidence intervals for each parameter. Figure 3 summarizes the ROC curves and corresponding AUC values. For candidate prioritization, we descriptively rank-ordered parameters by AUC point estimate (with patient-clustered 95% CIs); no formal pairwise AUC comparisons were performed.

In the pooled analysis (Fig. 3a), vBMD showed an AUC point estimate of 0.76 [95% CI, 0.55–0.90] and FEA-derived FL of 0.73 [0.52–0.90]; the selected texture features (GLN, HGLRE, SRHGLE, LRHGLE) ranged from 0.70 to 0.72 [CIs 0.48–0.86]. Confidence intervals overlapped across parameters; therefore, ordering and ranking based on AUC point estimates alone are not meaningful in this dataset and are presented for descriptive purposes only.

In the lumbar vertebrae subset (Fig. 3b), vBMD reached 0.94 [0.72–1.00], FEA-derived FL 0.82 [0.49–1.00], and texture features 0.72–0.75 [0.38–0.97]. Applying discrete lumbar thresholds yielded discrimination performances of 0.76 at the osteoporosis cutoff of 80 mg/mL and 0.69 at 120 mg/mL (osteopenia).

In the thoracic subset (Fig. 3c), vBMD had an AUC of 0.69 [0.41–0.87]. Applying cutoffs of 120, 100, and 80 mg/mL reduced the AUC points estimate to 0.68, 0.50, and 0.52, respectively. FEA-derived fracture load reached an AUC of 0.64 [0.38–0.85], and the selected texture features ranged from 0.66 to 0.71 [CIs 0.40–0.87].

Across pooled and region-stratified analyses, AUC point estimates varied numerically, but confidence intervals were wide and overlapping. In the absence of formal pairwise AUC testing, these patterns are descriptive, illustrative, and intended only for candidate prioritization.

## 4 Discussion

The present study aimed to determine whether vertebra-specific vBMD, FEA-derived FL, and CT-based TA features offer complementary value for discriminating incident vertebral fractures and to assess whether discrimination differs between the lumbar and thoracic regions. Our main findings were as follows. First, vBMD had the numerically largest AUC point estimates in this dataset (AUC = 0.76 [0.55–0.90] overall; 0.94 [0.72–1.00] for the lumbar region; 0.69 [0.41–0.87] for the thoracic region). Second, FEA-derived fracture load yielded AUC-based discrimination performance with numerical point estimates similar to those of vBMD (AUC = 0.73 [0.52–0.90] overall; 0.82 [0.49–1.00] for the lumbar spine; 0.64 [0.38–0.85] for the thoracic spine), with confidence intervals overlapping those of vBMD, and point estimates suggesting lower discrimination in the thoracic region for both measures. Third, selected texture features (GLN, HGLRE, SRHGLE, LRHGLE) demonstrated moderate discrimination (AUCs 0.66–0.75) with similar point estimates between the lumbar and thoracic subsets (0.75 in the pooled/lumbar versus 0.71 in the thoracic), and overlapping confidence intervals. No formal pairwise tests of AUC differences or equivalence were performed given the modest cohort size; these patterns are therefore descriptive and intended to prioritize candidates for future validation. Taken together, these exploratory results suggest that vertebral fracture discrimination in routine CT may, in the future, benefit from multivariable approaches that incorporate density, strength, and texture measures and consider lumbar and thoracic regions separately. In the present study, the AUC estimates are based on univariable analyses and do not establish definitive superiority of any single metric. Our intent is to develop a patient- and vertebral level-specific risk assessment derived from routine CT scans without additional radiation; the present analysis is hypothesis generating and not intended for immediate clinical application.

The discriminatory performance of the vBMD, particularly in the lumbar spine, is consistent with prior reports and extends them to a vertebra-level analysis [4, 40]. Our whole-spine AUC (0.76) closely matches the pooled AUC estimate of 0.77, as reported in a recent meta-analysis by Praveen et al. [40], while our lumbar AUC point estimate (0.94) was numerically higher than some prior single-level values reported in elderly men (0.83, measured at L1/L2 [22]) and mixed cohorts (0.815 at L3, [21]), but the wide confidence interval reflects the small sample size and indicates that these differences should be interpreted with caution. Measuring vBMD at each vertebral level rather than relying on a single “index” level likely captures local variations in bone density that contribute to mechanical failure, despite fractures occurring throughout the spine, highlighting the benefit of evaluating vBMD at each spinal level individually. However, applying the standard guideline cutoff values (80 and 120 mg/mL [38]) was associated with a marked drop in discriminatory performance (AUC), echoing clinical observations that up to half of patients with vertebral fractures are not classified as osteoporotic by DXA [41, 42]. These data underscore the limitations of fixed, population-based cutoffs, considering the prevalence of spatially heterogeneous bone loss.

In the thoracic spine, AUC point estimates for all parameters were lower than in the lumbar region, but the corresponding confidence intervals overlapped. Our thoracic vBMD AUC (0.69) is consistent with that of Ramschütz et al. (AUC of 0.70 for distinguishing subjects with and without prevalent thoracic fractures) [43]. However, when we imposed density thresholds, whether those were originally defined for lumbar vertebrae (80 mg/mL for osteoporosis and 120 mg/mL for osteopenia [38]) or the thoracic-specific proposal of 100 mg/mL by Rühling et al. [39], discriminatory performance deteriorated sharply. Specifically, the AUC dropped to 0.52 with the standard osteoporosis cutoff and to 0.50 with the criterion defined by Rühling et al. [39], while the osteopenia cutoff produced only a modest decline (AUC = 0.68). These results align with the observations from the lumbar subcohort and may suggest that fixed, population-derived cutoffs fail to adequately capture spatially heterogeneous bone loss. By contrast, the FEA-derived FL paralleled vBMD findings, with similar AUC point estimates (AUC 0.73 vs 0.76), rising to 0.82 (vBMD 0.94) in lumbar vertebrae and dropping to lower point estimates in the thoracic vertebrae 0.64 (vBMD 0.69), consistent with its reliance on the same CT-based density field. Considering the confidence intervals overlap substantially, our interpretation of these apparent differences is descriptive in nature. Collectively, these exploratory observations support the concept that region-specific parameters may be preferable and that lumbar thresholds should not be directly transplanted to the thoracic spine without further validation.

In our dataset, the FEA-derived FL showed lumbar AUC point estimates that were similar to those of vBMD and among the higher values across the investigated parameters, which is consistent with its biomechanical relevance. Similar AUC values have been reported by Allaire et al. (AUC = 0.80) [21], Wang et al. (AUC = 0.83) [22], and a pooled estimate of 0.80 was summarized by Praveen et al. [40]. Furthermore, FEA-derived FL integrates 3D geometry and non-linear material properties, capturing load-sharing phenomena that density alone cannot. In our data, the thoracic FEA-estimated FL AUC point estimate (0.64) was lower than its lumbar value (0.82), whereas Johannesdottir et al. reported level-invariant discrimination [23]. One possible explanation is that our vertebra-specific modeling is more sensitive to local anatomical factors, such as thinner endplates, stiffer rib cage constraints, or level-dependent disc health, which are not reproduced by standard axial compression loading and boundary conditions. Future models incorporating subject-specific loading vectors or ribcage constraints may improve thoracic discrimination models [44].

Texture analysis (TA) yielded modest discrimination across spinal levels. Selected higher-order gray-level run-length texture features (HGLRE, SRHGLE, LRHGLE, and GLN) had AUC point estimates of 0.66–0.72 (0.38–0.97), similar in magnitude to the best texture result (AUC = 0.75) reported by Dieckmeyer et al. [20] in a different cohort and protocol. Interestingly, AUC point estimates of these high-gray-level-run-length texture features changed little across regions (0.72 in the pooled and lumbar analyses versus 0.71 in the thoracic subset), with overlapping confidence intervals. This observation is speculative and generates the hypothesis that these parameters may capture aspects of trabecular microarchitecture that are less anatomical region-dependent than density or geometry. Given the limited sample size of the region-stratified analysis, this hypothesis cannot be tested in the present study and warrants confirmation in larger, independent cohorts. In our dataset, the similar AUC point estimates of these texture features across lumbar and thoracic levels are physiologically plausible and suggest that they could be considered as candidates for inclusion in future multivariable risk models, for example in combination with vBMD or FEA-derived FL.

These exploratory findings suggest that future vertebral-level fracture risk assessment from routine CT scans may benefit from multivariable models that combine vBMD, FEA-derived fracture load, and texture features; however, such multivariable models were not developed or evaluated in the present study.

### Limitations

In interpreting the results of our study, limitations must be taken into consideration. In our dataset of 420 patients, only 19 had an incidental fracture in the follow-up scan, and we needed to exclude 8, resulting in 11 patients and 20 fractured vertebrae. Thus, the primary limitations of this study relate to the small number of fracture patients and the resulting within-patient clustering of vertebrae. This small number of incidental fractures may limit power, especially for thoracic versus lumbar contrasts, and precludes external validation. Additionally, several patients contributed more than one fractured vertebra, introducing within-patient correlation that reduces the effective sample size. This is corroborated by the wide and overlapping confidence intervals calculated with the patient-clustered bootstrap method. Due to these constraints, we limited our analyses to univariable discrimination and did not develop multivariable discrimination models, establish operating points, or define management rules. The current results are, therefore, hypothesis-generating.

### Implications for translation

The small sample size and predominance of mild fractures (i.e., Genant grade 1) limit the precision of estimates and preclude robust subgroup analyses. Although vertebral- and sex-level matching with alignments regarding age and follow-up intervals were used, residual confounding from unmeasured factors (systemic therapies, comorbidities, and spinal degeneration) cannot be excluded. Translation into practice will require validation of candidate thresholds in independent cohorts and an assessment of how well discrimination risks agree with observed fractures. At present, no universally accepted clinical thresholds exist for FEA-derived FL or TA features, and any cutoff points are likely to depend on scanner and protocol because values vary with reconstruction kernel, voxel size, dose, and region-of-interest placement. If thresholds are pursued, they should be derived and validated in external cohorts and linked to age- and sex-specific categories or clinical fracture-risk levels rather than raw feature values. Our ROC analyses address the discrimination of incident fractures but not absolute risk; therefore, future work should develop and validate time-to-event or other calibrated risk models. While the clinical significance of Genant grade 1 deformities is more limited than for higher grades, their identification remains relevant for risk stratification; accordingly, we interpret our results as exploratory and prioritize confirmation in larger cohorts with more grade 2 and 3 fractures.

Because most scans were obtained for oncologic staging and detailed cancer/treatment information was unavailable, generalizability to non-oncologic populations may be limited. In this context, systemic therapies could influence bone quality independently of osteoporosis. External validation in broader, non-oncologic cohorts is warranted. The use of vertebral levels outside L1–L3 limits the direct applicability of American College of Radiology (ACR) thresholds [38]; any comparisons to ACR cut-points are contextual only and require level-specific calibration in future work. Although cases and controls were matched on sex, the number of fractured vertebrae per sex was small (men *n* = 8; women *n* = 12), which precluded sex-stratified ROC analysis in this dataset. We added sex-specific, exploratory ROC curves (Supplementary Figures S3–S4) and sex-specific descriptive summaries (Supplementary Table S2). Given the small per-sex sample sizes, estimates may be imprecise, and primary ROC results remain pooled over sex. In the future, adequately powered studies should examine sex-specific operating points and thresholds, in line with recent evidence that fracture discriminatory ability and cut-points may differ by sex (shown for hip/DXA by Wáng et al. [12]). Excluding patients with baseline prevalent vertebral fractures narrows the study spectrum and limits generalizability to individuals without prior vertebral fractures. Menopausal status was not recorded in this retrospective cohort; nonetheless, all women were ≥ 60 years, consistent with a postmenopausal age range. Because this was a retrospective study of non-trauma CT examinations performed for oncologic surveillance, a detailed recent fall history was not uniformly available; therefore, although fractures were identified in a non-trauma imaging context, the etiology of fractures cannot be established with absolute certainty. Given the oncologic setting, we excluded vertebrae with imaging suspicion of metastasis, but occult metastatic involvement cannot be entirely excluded by clinical routine CT. Additionally, CT-derived vBMD is not directly interchangeable with DXA-based values, complicating clinical translation. Because this retrospective cohort lacked paired non-contrast CT or DXA scans, we could not perform within-cohort calibration/validation of the contrast correction. We therefore relied on externally validated phase-aware methods and prior reports quantifying the effects of contrast on vBMD [7–10], [28]. We analyzed the discrete AUC values based on established cutoff values for vBMD to diagnose osteoporosis. This resulted in a drastic reduction of the AUC, highlighting, on the one hand, the room for improvement in determining fracture risk. Finally, the FE workflow, while largely automated, remains computationally intensive. In this context, cloud-based solvers and streamlined segmentation and geometry processing tools will be essential for routine clinical-level deployment. Further work should therefore focus on prospective studies that combine DXA, QCT, and clinical risk factors, refine subject-specific FE boundary conditions, particularly for the thoracic spine, and develop interpretable frameworks capable of integrating density, strength, and texture into a single vertebra-level risk score. Such efforts could potentially move the field closer to a personalized, mechanism-based fracture discrimination method to overcome the current limitations of using areal BMD alone in clinical settings.

## 5 Conclusion

In this exploratory, hypothesis-generating study, vertebra-specific vBMD, FEA-derived fracture load, and selected texture features each demonstrated measurable discrimination of incidental vertebral fractures in routine contrast-enhanced CT in univariable analyses. AUC point estimates varied numerically across parameters and regions, but wide and overlapping confidence intervals preclude meaningful ranking or confirmatory interpretation. Applying established vBMD thresholds for osteoporosis reduced AUC compared with continuous vBMD, illustrating the limitation of using fixed thresholds in this cohort. FEA-derived FL and selected texture features therefore represent additional candidate measures for future multivariable modeling to determine whether they provide complementary information beyond density alone. The observed numerical patterns in region-stratified analyses generate hypotheses regarding potential region-specific evaluation, but cannot be tested within the present dataset. Given the small number of fractures and the univariable design, these findings should be regarded as proof of concept; clinical application is premature. Future work requires confirmation in larger, diverse cohorts and the development of calibrated multivariate models with validated thresholds before informing decision-making.

## Supplementary Information

Below is the link to the electronic supplementary material.

ESM1 (PDF 590 KB**)**

## Data Availability

The data that support the findings of this study are available from the corresponding author upon reasonable request.
