# Supplementary material for: Vertebral-level discrimination of incidental vertebral fractures using volumetric BMD, texture features, and finite element–derived fracture load: an exploratory study
Source: Skeletal Radiol. 2026 Feb 16;55(6):1351–63. doi: 10.1007/s00256-026-05147-w (PMC13139215; doi:10.1007/s00256-026-05147-w)
Supplement: Supplementary file 1 — (PDF 590 KB) [file 256_2026_5147_MOESM1_ESM.pdf]

Supplementary Material to Manuscript title: “Vertebral-level discrimination of incidental vertebral fractures using volumetric BMD, texture features, and finite element–derived fracture load: an exploratory study”

S1: Height Measurements of Fractured Vertebrae

ID	Vertebra Level	Maximum Height	Minimum Height	Vertebra Level	Maximum Height	Minimum Height	Vertebra Level	Maximum Height	Minimum Height
1	**T8**	26,8	17,4	**T11**	29,2	21,9			
2	**T12**	25,8	9,1	**L1**	27,1	13,1			
3	**L1**	25,5	13,5						
4	**T6**	21,6	11,6						
5	**L1**	26,5	8,6	**L2**	26,7	11,5			
6	**T11**	25,6	13,5						
7	**L2**	24,8	13,4	**L3**	25,8	13,9	**L4**	22,8	13,7
8	**T12**	25,4	14,8	**L1**	27,7	17,5	**L2**	28,8	7,1
9	**T7**	19,4	10,2						
10	**T10**	22,7	17,1	**T11**	24,1	9,5	**T12**	22,3	5,6
11	**T12**	26,7	14,6						

S2: Descriptive statistics (average) stratified by fracture status and sex

	Women		Men	
	Fracture group	Control group	Fracture group	Control group
Number of Vertebrae	12	36	8	24
Age (years)	69,50	68,69	59,88	60,13
Follow Up (Months)	17,75	17,72	26,75	25,83
BMD	82,3	128,3	96,2	110,3
Fracture Load [N]	2227	3533	3229	3665
Variance	47,46	45,71	57,80	57,93
Skewness	0,94	1,28	1,26	1,10
Kurtosis	2,72	4,34	3,21	3,93
Energy	1,03E-04	7,02E-05	8,64E-05	9,76E-05
Contrast	1144,14	2078,09	1263,97	1228,82
Entropy	13,89	14,50	14,17	14,08
Homogeneity	0,05	0,04	0,04	0,05
Correlation	0,63	0,68	0,68	0,69
Sum Average	7,65E-04	6,62E-04	6,15E-04	6,67E-04
Variance	8,49E-03	1,08E-02	9,36E-03	8,23E-03
Dissimilarity	24,87	32,18	26,46	25,25
SRE	0,99	0,99	0,99	0,99
LRE	1,04	1,03	1,04	1,04
GLN	8,06E-03	6,26E-03	7,26E-03	7,40E-03
RLN	0,97	0,98	0,98	0,97
RP	0,99	0,99	0,99	0,99
LGLRE	1,79E-04	1,80E-04	1,49E-04	1,61E-04
HGLRE	19079,35	36207,99	21075,88	23620,92
SRLGLE	1,78E-04	1,79E-04	1,48E-04	1,60E-04
SRHGLE	18918,43	35996,37	20917,05	23429,33
LRLGLE	1,83E-04	1,83E-04	1,52E-04	1,65E-04
LRHGLE	19743,70	37073,32	21728,14	24410,99
GLV	0,06	0,07	0,05	0,05
RLV	3,83E-06	2,90E-06	3,16E-06	3,32E-06

S3: Sex stratified ROC curves women

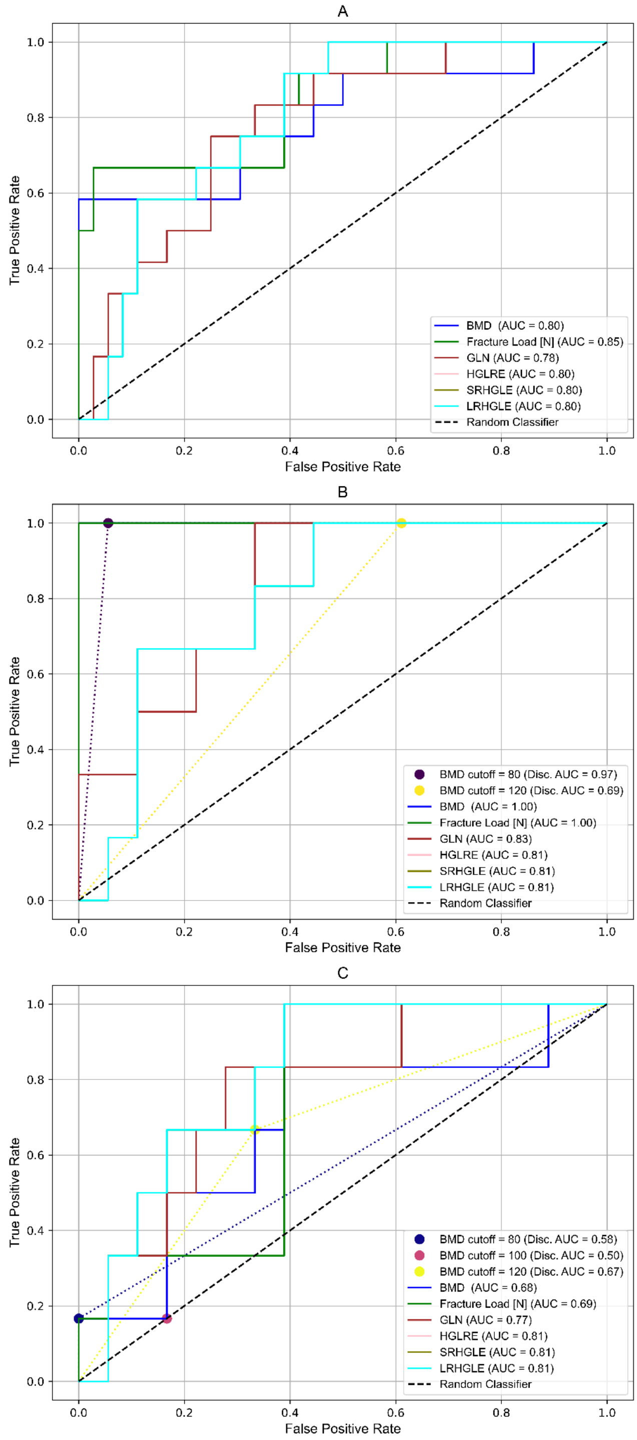

Figure 1 Exploratory sex stratified ROC analysis for women, ***a*** *all vertebrae (fractured n=12),* ***b*** *lumbar (fractured n=6) and* ***c*** *thoracic (fractured n=6)*

S4: Sex stratified ROC curves men:

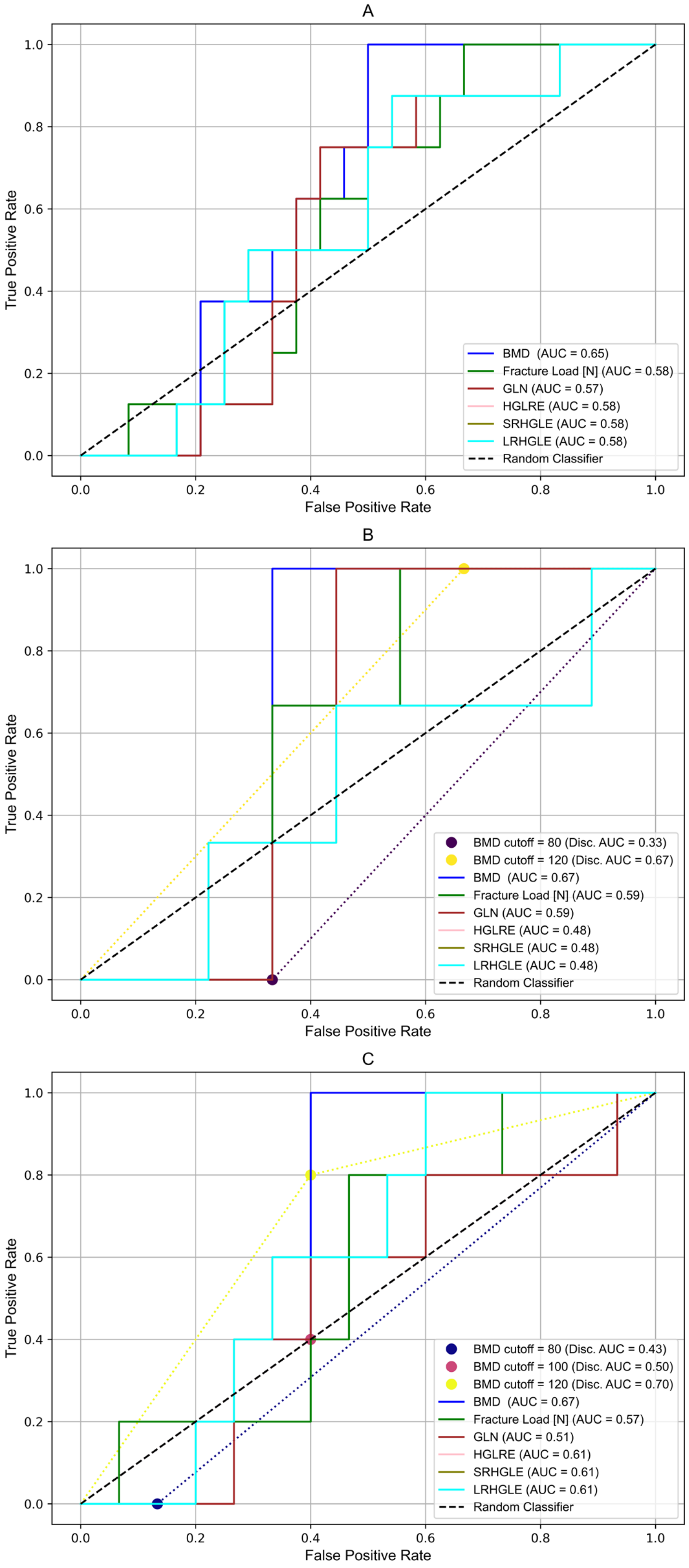

*Figure 2 Exploratory sex stratified ROC analysis for men, **a** all vertebrae (fractured n=8), **b** lumbar (fractured n=3) and **c** thoracic (fractured n=5)*
